# Remaining challenges in cellular flavin cofactor homeostasis and flavoprotein biogenesis

**DOI:** 10.3389/fchem.2015.00030

**Published:** 2015-04-22

**Authors:** Teresa A. Giancaspero, Matilde Colella, Carmen Brizio, Graziana Difonzo, Giuseppina M. Fiorino, Piero Leone, Roderich Brandsch, Francesco Bonomi, Stefania Iametti, Maria Barile

**Affiliations:** ^1^Dipartimento di Bioscienze, Biotecnologie e Biofarmaceutica, Università degli Studi di Bari Aldo MoroBari, Italy; ^2^Institut für Biochemie und Molekularbiologie, Universität FreiburgFreiburg, Germany; ^3^Dipartimento di Scienze per gli Alimenti, la Nutrizione e l'Ambiente, Università degli Studi di MilanoMilano, Italy; ^4^Dipartimento di Scienze della Vita, Istituto di Biomembrane e Bioenergetica, CNRBari, Italy

**Keywords:** FAD, FAD synthase, flavinylation, lysine specific demethylase 1, dimethylglycine dehydrogenase, rat, nucleus, mitochondria, nutri-epigenetics

## Abstract

The primary role of the water-soluble vitamin B_2_ (riboflavin) in cell biology is connected with its conversion into FMN and FAD, the cofactors of a large number of dehydrogenases, oxidases and reductases involved in a broad spectrum of biological activities, among which energetic metabolism and chromatin remodeling. Subcellular localisation of FAD synthase (EC 2.7.7.2, FADS), the second enzyme in the FAD forming pathway, is addressed here in HepG2 cells by confocal microscopy, in the frame of its relationships with kinetics of FAD synthesis and delivery to client apo-flavoproteins. FAD synthesis catalyzed by recombinant isoform 2 of FADS occurs via an ordered bi-bi mechanism in which ATP binds prior to FMN, and pyrophosphate is released before FAD. Spectrophotometric continuous assays of the reconstitution rate of apo-D-aminoacid oxidase with its cofactor, allowed us to propose that besides its FAD synthesizing activity, hFADS is able to operate as a FAD “chaperone.” The physical interaction between FAD forming enzyme and its clients was further confirmed by dot blot and immunoprecipitation experiments carried out testing as a client either a nuclear lysine-specific demethylase 1 (LSD1) or a mitochondrial dimethylglycine dehydrogenase (Me_2_GlyDH, EC 1.5.8.4). Both enzymes carry out similar reactions of oxidative demethylation, in which tetrahydrofolate is converted into 5,10-methylene-tetrahydrofolate. A direct transfer of the cofactor from hFADS2 to apo-dimethyl glycine dehydrogenase was also demonstrated. Thus, FAD synthesis and delivery to these enzymes are crucial processes for bioenergetics and nutri-epigenetics of liver cells.

## Introduction

The crucial role of the water soluble vitamin B_2_ or riboflavin (Rf) in cell metabolism is linked to Rf conversion into the enzyme cofactors flavin mononucleotide (FMN) and flavin adenine dinucleotide (FAD). In all the prokaryotic and eukaryotic cells the flavin cofactors ensure the functionality of hundreds of different flavoenzymes having dehydrogenase, oxidase, monooxygenase or reductase activities, and playing crucial roles in bioenergetics, photochemistry, bioluminescence, redox homeostasis, chromatin remodeling, DNA repair, protein folding, apoptosis, along with other physiologically relevant processes (Joosten and van Berkel, 2007). Thus, it is not surprising that deficiency of FAD-dependent enzyme and/or impairment of flavin homeostasis in humans and model animals have been linked to several diseases, such as cancer, cardiovascular diseases, anemia, abnormal fetal development, neuromuscular and neurological disorders (for rev. see Barile et al., 2013 and Refs therein). Therefore, understanding the membrane trafficking, homeostatic control, compartmentalisation, and turnover of Rf-derived flavin coenzymes within the cell is crucial to clarify the mechanism underlying the generation and maintenance of a normal cellular flavoproteome and of cellular metabolism. Some remaining challenges in cellular homeostasis of flavin cofactors and mitochondrial flavoprotein biogenesis will be dealt on in this paper.

Mammals must obtain Rf from the diet and, to a lesser extent, from intestinal microflora, whereas bacteria, protists, fungi, plants, and some animals can synthesize Rf from GTP and ribulose 5-P (Bacher et al., 2000). Dietary riboflavin is taken up in the human gastrointestinal tract by recently identified transporters, namely riboflavin transporters 1 (hRFT1) and 2 (hRFT2), that allow for vitamin concentration in the plasma and in blood cells (Haack et al., 2012). A third member of the Rf transporter family, namely riboflavin transporter 3 (hRFT3), is highly expressed in the brain (Yao et al., 2010; Patel et al., 2012; Foley et al., 2014). Mutations in hRFT2 and hRFT3 have been identified as in several individuals with a rare neurological disorder named Brown-Vialetto-Van Laere syndrome (Haack et al., 2012; Nabokina et al., 2012; Srour et al., 2014).

**Figure 1:**
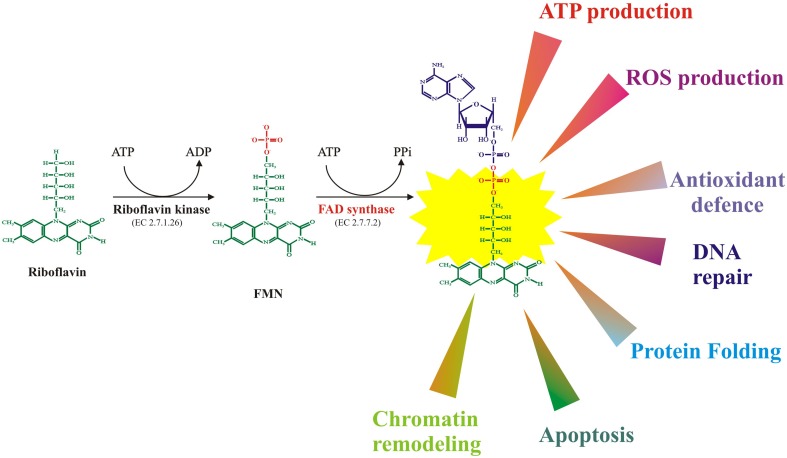
**FMN and FAD synthesis from riboflavin and main biological functions of flavoenzymes in mammalian cells**.

Once internalized in the cells, Rf conversion to cofactors occurs in two obligatory and ubiquitous steps, as schematised in Figure 1. The first enzyme of this pathway is riboflavin kinase (RFK, ATP: riboflavin 5′ phosphotransferase, EC 2.7.1.26), which transfers to Rf a phosphoryl group from ATP and forms FMN); the second enzyme—namely FAD synthase or FMN adenylyl transferase (EC 2.7.7.2, FADS or FMN-AT), previously known as FAD synthetase—is the enzyme responsible for FMN adenylation to FAD.

Compartmentalisation of flavin cofactor pools, generating the organelle-specific flavoproteome remains a question far from being elucidated and is sometimes a matter of debate (Lin et al., 2009; Barile et al., 2013; Kim and Winge, 2013; Lienhart et al., 2013). The main problem impairing an exhaustive comprehension of this issue derives from the necessity to coordinate FAD formation activities with events of cofactor assembly to client apo-proteins, that in eukaryotes occur mainly in mitochondria, but also in other compartments (Tu et al., 2000; Thorpe et al., 2002; Joosten and van Berkel, 2007; Stojanovski et al., 2008).

Subcellular localisation of FAD forming enzymes in mammalian, yeast and plant cells (Barile et al., 1993, 2000; Pallotta et al., 1998; Giancaspero et al., 2009) has been recently addressed at the molecular level (Bafunno et al., 2004; Torchetti et al., 2010; Liuzzi et al., 2012). These studies demonstrated that mitochondria possess their own FADS isoforms. In humans two different isoforms of human FADS were characterized, that are the products of alternative splicing generated from the human *FLAD1* gene (Brizio et al., 2006; Galluccio et al., 2007). Isoform 1 and 2 are located in mitochondria and cytosol, respectively (Torchetti et al., 2010).

Moreover, a novel FADS localisation was found to be the nucleus of mammalian cells (Giancaspero et al., 2013). Additional sub-cellular localisations for FADS cannot be ruled out, and they could contribute to the maintenance of distinct flavin cofactor pools in different cellular compartments. The existence of a FAD forming pathway in the nucleus appears in line with the proposal of a possible control of FAD availability on the activity of the nuclear enzyme lysine-specific demethylase-1 (LSD1), which carries out the demethylation of di- and mono-methyllysine 4 in histone H3, an important epigenetic modification (Luka et al., 2011, 2014; Hino et al., 2012).

A second series of questions about the cellular biochemistry of Rf derived cofactors concerns the mechanism of release of the newly-synthesized FAD to “client” flavoproteins (Torchetti et al., 2011; Miccolis et al., 2014). All the recombinant FADSs produced up to now exhibit the ability to bind FAD—the product of their activity—tightly but not covalently (1 mol FAD: 1 mol monomer), thus eliciting a typical flavoprotein absorbance spectrum, see Torchetti et al. (2011). Following SDS-PAGE the purified proteins still retain flavin fluorescence on the denaturing gel (Torchetti et al., 2011). FAD release from FADS is likely to be tightly controlled, and presumably requires well-defined conditions, including a correct redox state (Miccolis et al., 2014), the presence of an apo-protein accepting the cofactor and—possibly—some accessory proteins as reported for inorganic (Bonomi et al., 2008; Ye and Rouault, 2010) and organic cofactors (Padovani and Banerjee, 2009) in some human proteins. The hypothesis of a role of accessory/acceptor proteins in FAD release is consistent with (and—to some extent—supported by) the extremely low turnover number (*k_cat_*) measured for the purified enzyme (Torchetti et al., 2011) as the isolated protein.

Experiments described here are aimed at (i) confirming the multiple sub-cellular localization of FADS in human cells; (ii) investigating in some detail the FADS kinetics; (iii) addressing the issue of FAD release to different “client” flavoprotein, differing in nature and strength of FAD linkage and sub-cellular localization. In each case we aim at demonstrating that a direct interaction between the FAD forming machinery and the client flavoprotein occurs, and that a direct cofactor transfer from the donor to the apo-protein acceptor occurs in a sort of “FAD-chaperoning” action played by FADS *per se*, without a third reactant. The involvement of additional factors *in vivo*—including Hsp60 and Hsp10—is also discussed.

## Materials and methods

### Materials

All chemicals were of analytical or highest available grade and, unless otherwise stated, were obtained from Sigma-Aldrich. Polyvinylidene difluoride (PVDF) Hybond-P, Chelating Sepharose Fast Flow, and DEAE-Sephacel were from Amersham Ge-Healthcare. Reagents for protein assay were from Bio-Rad. Mouse anti-Hsp60 and Hsp-10 monoclonal antibodies were from Stressgen. Monoclonal mouse anti-LSD1 antibody was from Santa Cruz Biotechnology. Monoclonal mouse anti-β-actin antibody was from Abcam. Peroxidase conjugated anti-rabbit and anti-mouse IgG secondary antibodies were from Thermo Scientific. Alkaline phosphatase conjugated anti-rabbit and anti-mouse IgG secondary antibodies were from Sigma-Aldrich. Alexa Fluor conjugated anti-rabbit or anti-mouse IgG secondary antibodies were from Molecular Probes.

### Cell immunofluorescence and confocal microscopy

Immunofluorescence experiments were performed essentially as described elsewhere (Bruni et al., 2012; Cardone et al., 2012). Briefly, cells seeded on glass coverslips were fixed with 4% formaldehyde for 20 min and washed with PBS. After permeabilization (0.1% Triton X-100 in PBS, 15 min) and blocking (0.1% gelatin in PBS, 1 h), cells were incubated with the specific anti-FADS rabbit antiserum (1:200 dilution). Nuclei were counterstained with Hoechst 2 μM 33658. After washing, coverslips were mounted on microscope slides and confocal images were captured with a Leica TCS SP5 confocal microscope (Leica Microsystems, Mannheim) using a 63X (N.A. = 1.32) oil immersion objective, a 100 mW Argon laser (488 nm line) and a 50 mW diode (405 nm) as in (Gerbino et al., 2012). Confocal images were analyzed using the software Fiji (Schindelin et al., 2012) as in Tonazzi et al. (2013).

### Purification of recombinant hFADS2 and rat Me_2_GlyDHs

Purified recombinant 6His-hFADS2 was prepared as described in Torchetti et al. (2011). Protein concentration and FAD/ protein monomer ratio (i.e., the flavinylation level) were estimated by absorbance spectra, as in Torchetti et al. (2011). Purified recombinant 6His-m-Me_2_GlyDH and 6His-p-Me_2_GlyDH were prepared in both their apo- and holo-form essentially as described in Brizio et al. (2004) and Brizio et al. (2008), respectively. The flavinylation level of purified recombinant Me_2_GlyDH was estimated by measuring the UV fluorescence of the SDS-PAGE separated protein band, due to covalently bound FAD cofactor, essentially as described in Brizio et al. (2004, 2008).

### Kinetic analysis of 6His-hFADS2

FAD synthesis rate was measured at 37°C in 1 mL of a standard reaction medium consisting of 50 mM Tris-HCl, 5 mM MgCl_2_, pH 7.5 in the presence of FMN and ATP added at the appropriate concentrations. The reaction was started with the addition of 6His-hFADS2. FAD synthesis rate was determined by taking advantage of the differential fluorimetric properties of FAD with respect to FMN (Barile et al., 1997). Fluorescence time courses (excitation at 450 nm and emission at 520 nm) were followed in a LS50 Perkin–Elmer spectrofluorimeter. In each experiment, FAD and FMN fluorescence were calibrated individually using standard solutions whose concentration was calculated by using ε_450nm_ = 12.2 mM^−1^·cm^−1^ for FMN and 11.3 mM^−1^·cm^−1^ for FAD. Under the experimental conditions used here, the FAD fluorescence constant (*K*_FAD_) was about 10 times lower than that of FMN (*K*_FMN_). Thus, the rate of FAD synthesis, expressed as nmol FAD min^−1^ (mg protein)^−1^, was calculated from the rate of fluorescence decrease, measured as the tangent to the initial part of the experimental curve by applying the equation described in detail elsewhere (Torchetti et al., 2011).

### Reconstitution of holo-DAAO activity

The reconstituted holo-D-amino acid oxidase (D-AAO, EC 1.4.3.3) activity, derived from FAD binding to the apo-D-AAO, was followed spectrophotometrically as described in Barile et al. (2000), using 25 mM D-alanine as substrate. NADH oxidation in the L-lactate dehydrogenase (LDH, EC 1.1.1.27)-coupled reaction was followed spectrophotometrically at 340 nm. The reaction rate was calculated by measuring the slope of the tangent to the linear part of the experimental curve. This rate was proven to be proportional to FAD concentration. Calibration curves were obtained by using standard FAD solutions.

### Preparation of pure nuclei from rat liver

Nuclei were isolated from the liver of male Wistar rats by differential centrifugation in sucrose gradient essentially as in Giancaspero et al. (2013). The isolated nuclei were finally resuspended in nuclear buffer (20 mM Tris-HCl, pH 7.5, 0.5 mM PMSF). The purity and functionality of the nuclear fractions were checked as in Giancaspero et al. (2013) by following (i) the increase in the enzymatic activity of the nicotinamide mononucleotide adenylyltransferase, a central player NAD biosynthesis, mostly expressed in the nucleus as isoenzyme 1 (Orsomando et al., 2012), (ii) the decrease in the enzymatic activity of LDH a marker enzyme of the cytosolic compartment. As a control immunoblotting assays of lamin A/C (a nuclear marker) and of tubulin (a cytosolic marker) were also carried out, as in Giancaspero et al. (2013).

### Preparation of mitochondrial matrix from rat liver and its fractionation

The mitochondrial matrix was obtained from purified rat liver mitochondria, as previously described (Barile et al., 1997) and fractionated by ionic-exchange chromatography according to Brizio et al. (2000) and Bafunno et al. (2004). Briefly, the mitochondrial matrix (17.5 mg/mL) was applied onto a DEAE-Sephacel column (2 cm × 0.7 cm), equilibrated with 50 mM Tris-HCl, pH 7.5. The column was washed with the starting buffer (1 mL), then eluted with a discontinuous gradient of NaCl (50–250 mM, step 50 mM) in the same buffer. For each step of the gradient two fractions of 0.5 mL were collected. Each chromatographic fraction was analyzed by western blotting using a rabbit polyclonal antiserum directed against human FADS (anti-FADS, International Application number PCT/IT2009/000062 filed February 23, 2009 by Barile, Torchetti, Indiveri, Galluccio), as described in Brizio et al. (2013). Flavinylation Stimulating Factor (mtFSF) activity was assayed in each fraction here by measuring the increase of the flavin fluorescence of Me_2_GlyDH protein band separated by SDS-PAGE.

### Immunoblotting

SDS-PAGE separated proteins were electro-transferred onto a PVDF membrane using a Trans-Blot semidry electrophoretic transfer cell (Sigma–Aldrich). The immobilized proteins were incubated overnight with a 3000-fold dilution of anti-FADS antiserum, as in Giancaspero et al. (2013). Other antibodies were used to reveal and quantify protein markers, including a mouse monoclonal anti-β-actin antibody (1:10,000 dilution), and mouse monoclonal antibodies: anti-LSD1 (1:1000 dilution); anti-Hsp60 (1:1000 dilution); anti-Hsp10 (1:1000 dilution). Bound antibodies were visualized with secondary anti-rabbit or anti-mouse IgG antibodies conjugated with peroxidase (1:3500 dilution) or with alkaline phosphatase (1:3500 dilution).

### Dot blot experiments

To identify the interaction between nuclear proteins and hFADS2, purified rat liver nuclei (50 or 100 μg) or purified recombinant 6His-hFADS2 (5 μg) were dot-blotted onto a nitrocellulose membrane. Where indicated, rat liver homogenate (homo, 50 or 100 μg), nuclei (50 or 100 μg) or nuclear buffer (none) were added to the dotted membrane. After 30 min incubation at 37°C the membranes were washed, saturated in blocking solution containing BSA 3–5% and probed with antibodies directed against human FADS (anti-FADS), LSD1 (anti-LSD1) or actin (anti-ACT1). Following a washing step, the bound antibodies were visualized with secondary anti-rabbit or anti-mouse IgG antibodies conjugated with alkaline phosphatase (1:3500 dilution). To reveal the interaction between Me_2_GlyDH and hFADS2, purified recombinant Me_2_GlyDH (1 μg) was dotted onto a nitrocellulose membrane. Purified recombinant 6His-hFADS2 (3 μg, in HEPES buffer 40 mM, pH 7.5, 5 mM MgCl_2_) was added to the dotted membrane in the presence of 5 mM ATP and, where indicated, of 20 μM FMN. After 30 min incubation at 37°C, the membranes were washed and probed with the anti-FADS antiserum as above.

### Immunoprecipitation experiments

Anti-FADS antiserum was added to a Dynabeads® Protein G immuno-precipitation kit according to manufacturer's procedure and used to immuno-precipitate purified rat liver nuclei (50 μg). The nuclear immuno-precipitated proteins were analyzed by immuno-blotting with the anti-FADS antiserum. The same PVDF membrane was tested with the anti-LSD1 and anti-ACT1 antibodies after performing a stripping procedure. The bound antibodies were visualized with secondary anti-rabbit or anti-mouse IgG antibodies conjugated with peroxidase (1:3500 dilution).

### Other assays

Protein concentration was assayed according to Bradford (1976), using bovine serum albumin (BSA), as standard. Quantitative evaluation of UV fluorescent and immuno-reactive protein bands was carried out by densitometric analysis using the *Image lab* software (BIORAD).

## Results and discussion

### Subcellular localization of FADS in human liver carcinoma (HepG2) cells

**Figure 2:**
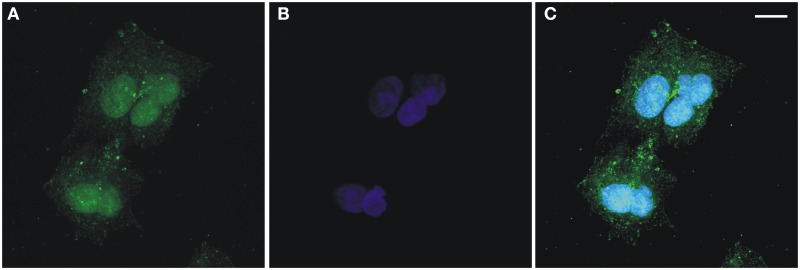
**Confocal microscopic analysis of hFADS subcellular localization in human liver carcinoma (HepG2) cells**. Maximum-intensity projections of confocal laser scanning image stacks of HepG2 cells labeled with the polyclonal anti-FADS antiserum followed by incubation with an Alexa Fluor 488-conjugated anti-rabbit antibody (green, **A**). Nuclei were stained with Hoechst 33658 (blue, **B**). The overlay of the two images is represented in **(C)**. Scale bar = 10 μm.

As stated in the Introduction, the first challenge of this paper is to provide further evidence of multi-compartmentalisation of FADS in human cells. The presence of FADS in both the mitochondrion and nucleus was demonstrated for the first time in freshly isolated rat liver fractions (Giancaspero et al., 2013). To confirm the multi-compartmentalisation of FADS in human cells, immunofluorescence experiments were carried out on a human cell line derived from liver carcinoma, i.e., HepG2 (Figure 2). After fixation, permeabilization and incubation with the anti-FADS antiserum the immuno-complexes were visualized with a secondary antibody conjugated with Alexa Fluor 488 (Figure 2A). After nuclei counterstaining with Hoechst 33568 (Figure 2B), the coverslips were analyzed by confocal fluorescence microscopy. As apparent in Figures 2A,C, besides the expected cytoplasmic localization, a clear FADS-immunoreactivity was visible in the nucleus of HepG2 cells.

### Steady state kinetics of recombinant FADS

**Figure 3:**
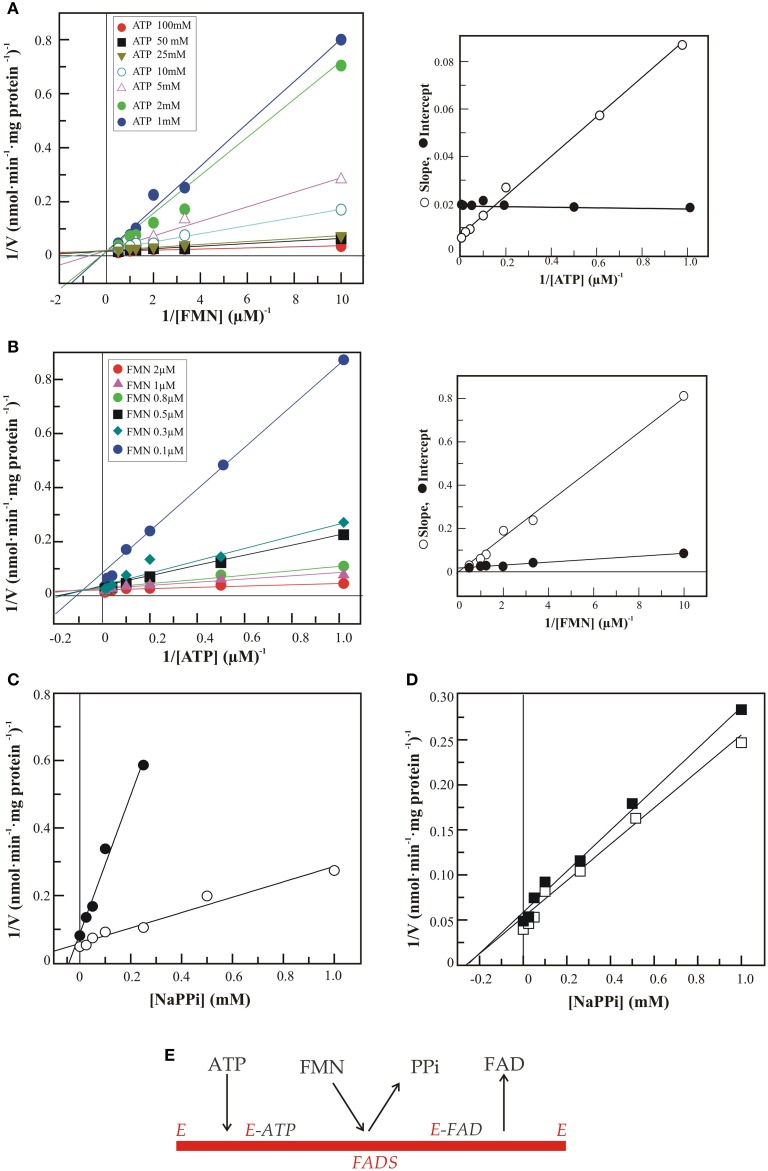
**Steady state kinetic analysis of FAD synthesis catalyzed by hFADS2**. FAD synthesis rate, catalyzed by 6His-hFADS2 (2 μg, 35.4 pmoli), was measured by the initial rate of fluorescence decrease (λ_ex_ at 450 nm, λ_em_ at 520 nm) at 37°C in 50 mM Tris-HCl, pH 7.5 in the presence of 5 mM MgCl_2_ and FMN and ATP at the reported concentrations. Where indicated, sodium pyrophosphate was added at the indicated concentrations. In **(A)** the initial rates represented by the Lineweaver–Burk plot of 1/ν vs. 1/[FMN] at fixed ATP concentrations. In the inset the slope and intercepts of the Linaeawever-Burk plot (with FMN as variable substrate) vs. 1/[ATP]. In **(B)** the initial rates represented by the Lineweaver–Burk plot of 1/ν vs. 1/[ATP] at fixed FMN concentrations. In the inset the slope and intercepts of the Linaeawever-Burk plot (with ATP as variable substrate) vs. 1/[FMN]. In **(C)** the Dixon plot of the inhibition by pyrophosphate at ATP 10 μM and FMN 0.3 μM (•) or 2 μM (◦). In **(D)** the Dixon plot of the inhibition by pyrophosphate at FMN 2 μM and ATP 10 μM (◼) or 50 μM (◻). In **(E)** the sequential ordered bi-bi mechanism for 6-His-hFADS2 is represented.

Figure 3 reports the results of steady state kinetic experiments carried out with the recombinant 6His-hFADS2, i.e., the cytosolic form of the enzyme (Torchetti et al., 2010), and aimed at addressing the second challenge, i.e., the enzyme mechanism of the synthase.

The dependence of the rate of FAD formation on FMN concentration (from 0.1 to 2 μM) at fixed concentrations of ATP was investigated by means of a rapid spectrofluorimetric assay (Barile et al., 1997). Results are reported according to a Linaeawever-Burk plot (Figure 3A). The dependence of reaction rate on ATP (ranging from 0 to 100 μM) at fixed FMN concentrations was investigated in parallel experiments (Figure 3B).

When FMN is varied at different ATP levels (from 1 to 100 μM), the pattern intersects on the vertical axis (Figure 3A). Thus, the *Km_FMN_* value decreases (from 8 μM to 0.4 μM) at increasing ATP concentration. Conversely, when ATP is varied, the patterns intersects in the forth quadrant (Figure 3B). Thus, *Km_ATP_* does not change significantly (at ≈ 15 μM) when increasing FMN concentration. A replot of the slopes of the Lineawever-Burk plot (with FMN as variable substrate) vs. 1/[ATP] has a finite vertical intercept (inset of Figure 3A). A replot of the slopes of the Lineawever-Burk plot (with ATP as variable substrate) vs. 1/[FMN] goes through the origin, instead of having a finite vertical intercept. This patterns are consistent with a “sequential ordered bi-bi” mechanism for 6-His-hFADS2, and allow to define the order of substrate binding to the enzyme as ATP binding prior to FMN.

To define the order of product release the inhibition patterns of PPi, one of the products in the FADS reaction, were studied according to a Dixon analysis (using fixed FMN concentration at 2 μM in Figure 3C and using fixed ATP concentration at 10 μM in Figure 3D). In Figure 3C a non-competitive inhibition pattern by PPi was observed when ATP is the variable substrate, since the patterns intersect on the horizontal axis. In Figure 3D the inhibition by PPi against FMN is consistent with competitive inhibition, since the pattern intersects in the forth quadrant. This mechanism is a special case of an ordered mechanism, named as the Theorell-Chance (see *The enzyme- Kinetics and mechanism*, Clealand, 1970). This allows to establish that PPi is the first product released immediately after FMN binds to the enzyme.

The results in Figure 3 clearly indicate that FAD synthesis occurs via an ordered bi-bi mechanism in which ATP binds prior to FMN, and pyrophosphate is released before FAD. A similar mechanism has been reported for other FADS, such as those from rat (Oka and McCormick, 1987) and *C. glabrata* (Huerta et al., 2009).

The slow release of FAD after PPi is consistent with (and to some extent supported by) the extremely low turnover number (*k_cat_*) measured for the recombinant purified enzyme when monitoring FAD synthesis by direct and indirect assays and the observation that FAD remains tightly bound to the recombinant purified enzyme (Torchetti et al., 2011). Thus, our data indicate that FAD release may represent the rate-limiting step of the whole catalytic cycle and that the processes leading to FAD synthesis and delivery to client apoproteins may be tightly controlled by factors others than FADS itself.

**Figure 4:**
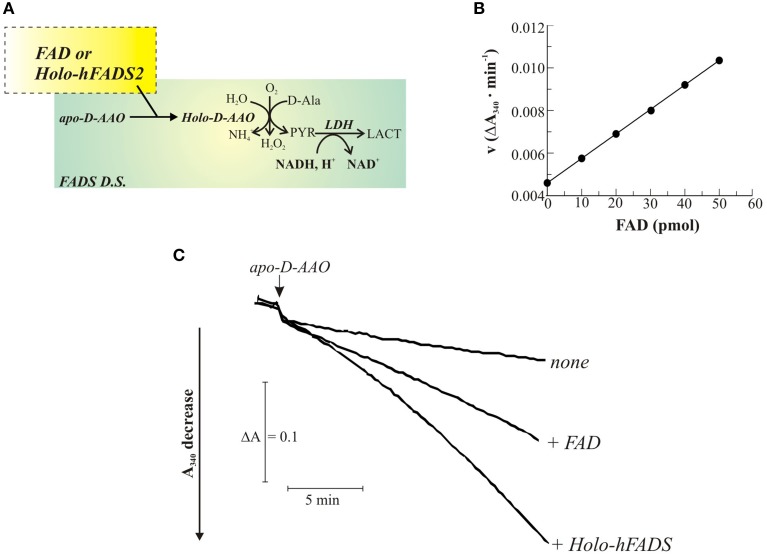
**FAD delivery from 6His-hFADS2 to the client apo-D amino acid oxidase**. The release of FAD from the purified recombinant 6His-hFADS2 to apo-DAAO was assayed enzymatically, as described in Materials and Methods and schematized in **(A)**, by measuring the activity of reconstituted holo-DAAO (derived from FAD binding to apo-DAAO). **(B)** Calibration curve obtained with a FAD standard. In **(C)** typical traces are shown. Apo-DAAO was added in the absence (none) or in the presence of purified 6His-hFADS2 (Holo-hFADS2, 1.2 μg, 20 pmol) or in the presence of commercial FAD (20 pmol) at 37°C in 100 μL of 50 mM Tris-HCl, pH 7.5. Reconstituted holo-DAAO activity was measured as described in Materials and Methods.

The ability of the recombinant enzyme to catalyze FAD formation and delivery to a client apo-flavoprotein was next followed by a continuous spectrophotometric assay in which newly synthesized FAD is used to reactivate apo-D-AAO as a client flavoprotein. The reconstituted activity of D-AAO on FAD incorporation is measured as the rate of NADH oxidation in a coupled reaction with LDH (Figure 4A). Due to the time-dependence of the reconstitution reaction (Casalin et al., 1991; Barile et al., 2000), the reaction rate increases with time, reaching a maximum constant value, that was demonstrated to be proportional to the amount of the externally added cofactor (Figure 4B). In the experiment reported in Figure 4C, apo D-AAO was incubated in the absence or presence of 1 μg 6His-hFADS2 (which binds 1 mol per monomer of FADS tightly and that corresponds to 20 pmol of the 56.5 kDa monomer and i.e., to 20 pmol of FAD bound to 6His-hFADS2) as well as in the presence of equimolar amount of free FAD (20 pmol). The rate of absorbance decrease following the addition of apo-D-AAO was equal to 0.0046 ΔA/min in the absence of FAD addition (trace *none*). This rate is presumably due to a small fraction of holo-enzyme present in the assay. When 20 pmol of FAD were added, the maximum value of the rate of absorbance decrease was equal to 0.0069 ΔA/min, in agreement with the occurrence of binding of FAD to apo-D-AAO. When adding 1 μg of 6His-hFADS to the assay, the rate of absorbance loss increased with time, reaching a rate two-fold higher than what measured with free FAD was added (ΔA/min = 0.0139). These observations are consistent with the apo-holo transition of DAAO being accelerated by 6His-hFADS. Therefore, FADS seemed to operate not only as a synthase but also as a FAD “chaperone, ” that is supposed to directly interact with the client apo-flavoprotein during holoenzyme formation.

**Figure 5:**
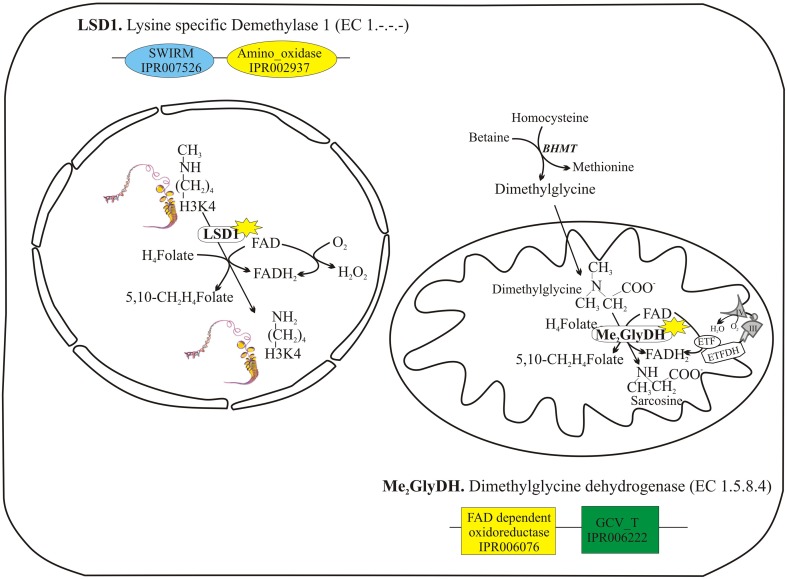
**Two client apo-flavoproteins: nuclear lysine specific demethylase 1 (LSD1) and mitochondrial dimethylglycine dehydrogenase (Me_2_GlyDH)**. LSD1 and Me_2_GlyDH are both FAD containing enzyme which carry out similar reactions of oxidative demethylation, assisted by tetrahydrofolate (H_4_Folate) used to form 5,10-methylene-tetrahydrofolate (5,10-CH_2_H_4_Folate).

A further question was whether the expected physical interaction between FAD forming enzyme and its client could occur also with apo-flavoproteins located both in the nucleus and in the mitochondrion (Figure 5), where the enzyme could play a primary role in cellular homeostasis. The occurrence of such an interaction was previously hypothesized by different groups (Barile et al., 2013; Brizio et al., 2013). To these scope, mitochondrial dimethylglycine dehydrogenase (Me_2_GlyDH, EC 1.5.8.4) and nuclear lysine specific demethylase 1 (LSD1, EC 1.-.-.-) were tested as client flavoproteins. Recombinant purified hFADS was used to test the interaction with these client proteins by means of dot-blot and immuno-precipitation experiments, and to verify transfer of the cofactor to the client apoprotein, wherever possible.

### LSD1 as a client flavoprotein

LSD1 is a nuclear FAD-containing amine oxidase, that catalyzes the demethylation of mono- and dimethylated Lys4 on histone H3, one of the most important recent discoveries in nutri-epigenetics (Hino et al., 2012). During the course of the reaction, FAD oxidizes the lysine N-methyl amine to lysine N-methylimine, and FADH_2_ is reoxidized to FAD by molecular oxygen producing hydrogen peroxide. In the absence of tetrahydrofolate, the oxidation of methyl groups generates formaldehyde, potentially highly toxic for the nucleus, that is expected to be reduced and recycled to give S-adenosyl methionine (SAM), the main nuclear methyl donor (Tyihak et al., 1998). Recently it has been demonstrated that LSD1, as well as Me_2_GlyDH, is able to bind tetrahydrofolate. Thus, both these reactions generate N-5,10-methylene-tetrahydrofolate (Figure 5) in a sort of oxidative demethylation of the substrates (Luka et al., 2011, 2014). Therefore, nuclear pool of flavins and folates seems to be strictly interconnected in controlling the crucial processes of methylation status of hystones and in nutri-epigenetics.

The LSD1 structure is well characterized. The protein consists of a classical FAD-amino oxidase domain (IPR002937) where the flavin cofactor is bound (not covalently). The folate-binding site is located in the active center in close proximity to FAD. The N-terminus contains a SWIRM domain (IPR007526), presumably responsible for nucleosome recognition (Da et al., 2006) and it is preceded by a disordered extension (Shi et al., 2004). Two isoforms are reported in Entrez Gene for humans, as products of alternative splicing of the *KDM1A* gene, the canonical isoform being a 852 residue long polypeptide (92.9 kDa). A single rat isoform - corresponding to LSD1—is reported in Entrez Gene, as a 872 residue long protein (Mr = 94.4 kDa) named *Kdm1a*, exhibiting a similarity of 98% with respect to human protein. Nevertheless, a wide BLAST search in non-redundant protein sequence databases using as a query the canonical human LSD1 isoform gave an additional product of 776 amino acids (86.1 kDa), exhibiting a 99% identity with the human protein. This isoform lacks of the first 96 aa respect to rat *Kdm1a* protein. A prediction made with PSORTII program, gives a score of 11% of nuclear localisation for Kdm1a and of 39% for the “similar to AOF2 protein.” *Kdm1a* scores a 44% probability for an extracellular localisation and a 22% probability for a cytosolic one. The “similar to AOF2 protein” has a high probability to be cytosolic (52%) and a very low probability to be localized in the mitochondrion (4%). No cleavage sites have been identified by this bioinformatics approach.

In order to get some insight into LSD1 biogenesis, a nuclear fraction was purified from rat liver homogenate, as previously described (Giancaspero et al., 2013) and the enrichment in specific nuclear protein assessed by measuring the increase in the enzymatic activity of the nuclear enzyme nicotinamide mononucleotide adenylyltransferase, as described under Materials and Methods.

**Figure 6:**
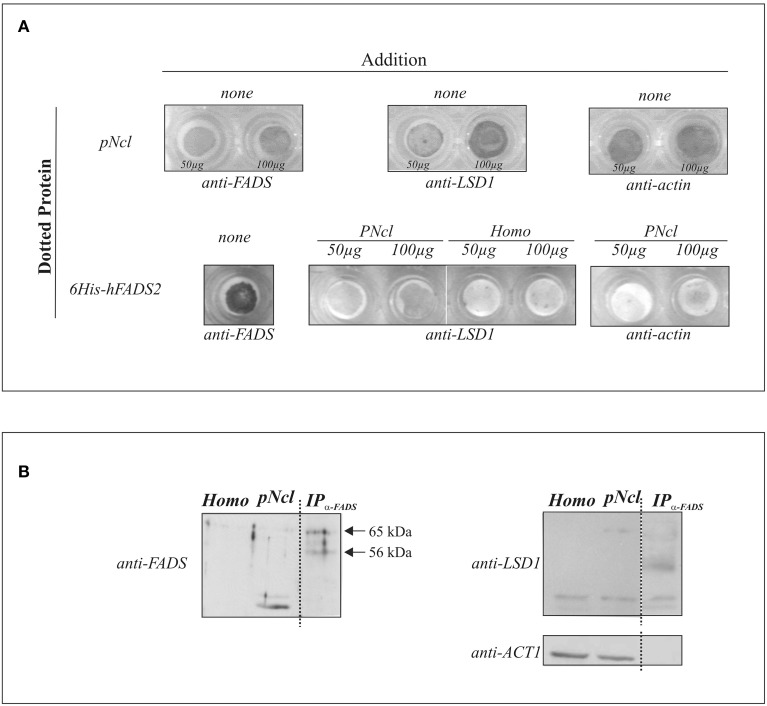
**FADS/LSD1 interaction as revealed by immunological techniques**. In **(A)** the dot-blot assay is reported: briefly, purified rat liver nuclei (pNcl, 50 or 100 μg) resuspended in the nuclear buffer or 6His-hFADS2 (5 μg) were dotted onto a nitrocellulose membrane. Where indicated pNcl (50 or 100 μg), homogenate (homo, 50 or 100 μg) or the nuclear buffer (none) were added to the dotted membrane. Protein/protein interaction was revealed immunochmically as described in Materials and Methods. In **(B)** rat liver homogenate (homo, 25 μg), nuclei (pNcl, 25 μg) and the immunoprecipitate (IP_anti−FADS_) from nuclear proteins (50 μg) were analyzed by immunoblotting with anti-FADS antiserum, as described in Materials and Methods. The same PVDF membrane was analyzed with the anti-LSD1 and anti-ACT1 antibodies after stripping procedure.

First of all, the existence of FADS in the nucleus was revealed by dot-blot, using a polyclonal antiserum against hFADS (anti-FADS) and increasing amount of nuclear protein (50 or 100 μg) (Figure 6A). In parallel runs, dotted nuclear proteins were tested for the presence of LSD1 by using a commercial monoclonal antibody. The identity of the nuclear fraction was further validated by observing the enrichment of the nuclear protein LSD1 with respect to the spots obtained when 50 or 100 μg of homogenate were added as a control (data not shown).

In order to investigate if the natural nuclear LSD1 protein is able to directly interact with FADS, in a parallel experiments 5 μg of recombinant human 6His-hFADS2 were dotted on the membranes, and successful dotting was tested by using the anti-FADS antiserum. FADS-dotted membranes were washed and incubated for 15 min with nuclear proteins, and the amount of nuclear LSD1 bound to 6His-hFADS in the dots measured immunochemically in comparison with the amount of protein bound when starting from the homogenate. No 6His-hFADS2 was present in controls, and the specificity of 6His-hFADS2 /LSD1 interaction was tested by verifying the absence of actin bound to the 6His-hFADS dotted membrane (Figure 6A).

To further validate the specificity of 6His-hFADS2/ LSD1 interaction, immuno-precipitation experiments were performed and reported in Figure 6B. Following over-night incubation (according to the manufacturer-suggested procedure, see below), different anti-FADS cross-reactive bands were found in subsequent electrophoretic analysis of the products. The most evident band was found at about 38 kDa, likely being a hydrolysis product (see also Giancaspero et al., 2013). In the same fractions, after stripping procedure, an anti-LSD1 reactive band migrating at 97 kDa was revealed (in good agreement with the molecular mass of the kdm1a isoform) accompanied by a lower migrating band (apparently a hydrolysis product of about 58 kDa). The 97 kDa band appeared significantly enriched in the nuclear fraction.

Purified nuclear fractions were, then, immuno-precipitated by using the anti-FADS antiserum and the immuno-precipitation kit Dynabeads Protein G. Immuno-precipitation resulted in a clear enrichment of the three main anti-FADS cross reacting bands located in the 65–56 kDa range, as expected from the predicted molecular mass of rat FADSs (Giancaspero et al., 2013). The same fraction was also revealed by using the anti-LSD1 antibody. Different cross reactive bands were immune-precipitated together with FADS, and one of these bands migrates at about 80 kDa. ESI-MS/MS analysis will be performed to identify whether the 80 kDa band is a hydrolytic product of kdm1a or rather the protein similar to AOF2. Thus, the emerging picture favors the proposal of a physical interaction between the nuclear apo-flavoprotein LSD1 and FADS, an interaction of high relevance for attachment of a FAD cofactor during enzyme biogenesis.

### Me_2_GlyDH as a client flavoprotein

The mechanism of flavoprotein biogenesis is of particular relevance in mitochondria, since they are the main site of localisation of flavoenzymes bearing both covalently-bound FAD and non-covalently bound FAD or FMN (Heikal, 2010; Barile et al., 2013; Lienhart et al., 2013). As far as the mitochondrial flavoproteome is concerned we focused our attention on Me_2_GlyDH, a key enzyme of folate one-carbon metabolism and choline catabolism (Figure 5), located in the mitochondrial matrix. In the presence of tetrahydrofolate (THF), Me_2_GlyDH catalyzes the oxidative demethylation of dimethylglycine to yield sarcosine and 5,10-methylene-THF. In the natural mature Me_2_GlyDH the FAD cofactor is covalently linked to the enzyme via a histidyl(N3)-(8α) FAD linkage occurring at His84 (Cook et al., 1985). Me_2_GlyDH is synthesized in the cytosol as a precursor protein containing an N-terminal extra-sequence, which is removed in the organelle by the mitochondrial processing peptidase (MPP) (Otto et al., 1996). The flavin attachment event *in vivo* occurs in mitochondria before removal of the pre-sequence (Brizio et al., 2002).

Both the mature (mMe_2_GlyDH) and the precursor (pMe_2_GlyDH) forms of rat Me_2_GlyDH were produced in *Escherichia coli* as, respectively, a N-terminally and C-terminally 6-His-tagged fusion protein (Brizio et al., 2004, 2008). Flavinylation of the *in vitro* synthesized apo-Me_2_GlyDH protein seemed to proceed spontaneously in the presence of FAD, in line with an autocatalytic process (Otto et al., 1996). Nevertheless, the rate of Me_2_GlyDH holoenzyme formation was found to be stimulated by protein factor(s) localized in mitochondrial matrix, that were tentatively named *mitochondrial flavinylation stimulating factor(s)* (mtFSF) (Brizio et al., 2000, 2002). mtFSF(s) elutes at 50 mM NaCl (DEAE50) following fractionation of the mitochondrial matrix by ionic-exchange chromatography on a DEAE-Sephacel column (Brizio et al., 2000, 2013).

**Figure 7:**
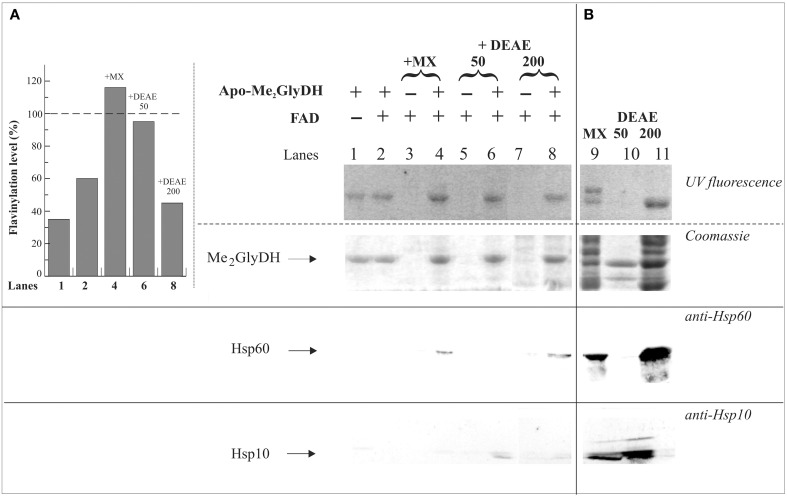
**Evidences of Me_2_GlyDH flavinylation and identification of possible interactors**. In **(A)** 6His-apo-Me_2_GlyDH (10 μg) was incubated at 37°C in flavinylation medium made of 50 mM Tris-HCl, pH 7.5, 5 mM MgCl_2_ 5 mM, 0.5% Triton X-100, 5 mM ATP, in the absence or presence of 20 μM FAD. Where indicated rat liver mitochondrial matrix (MX) (lane 4) or MX fractions eluted onto a DEAE-Sephacel column with 50 and 200 mM NaCl (D50 and D200 fractions, 100 μg each, lanes 6 and 8, respectively) were added to the reaction mixture. As a control the same matrix and DEAE fractions were incubated in the same experimental condition in the absence of apo-Me_2_GlyDH and in the presence of 20 μM FAD (lanes 3, 5, 7). After 1 h incubation, each sample was passed on a Ni-Chelating Sepharose to re-isolate the recombinant 6His-apo-Me_2_GlyDH and possible interactors. After washing with 50 mM imidazole, bound 6His-apo-Me_2_GlyDH and its possible interactors were eluted with 500 mM imidazole, precipitated with acetone, and analyzed by SDS-PAGE. The flavin fluorescence of SDS-PAGE separated proteins was visualized by UV irradiation of the unstained gel soaked in 10% acetic acid. Protein bands were then stained with Coomassie Brilliant Blue. The interactors were searched for by immunoblotting analysis carried out using anti Hsp60 and Hsp10 antibodies. The flavinylation level of Me_2_GlyDH (inset) was estimated through image analysis as described in Materials and Methods. In **(B)** matrix and fractions D50 and D200 (100 μg each, lanes 9–11) from ion-exchange chromatography were analyzed by SDS-PAGE and immunoblotting as described in **(A)**.

A simple, rapid and direct method for determining the flavinylation level of the Me_2_GlyDH consists in revealing holoenzyme flavin fluorescence upon irradiation with UV light of SDS-PAGE gels provides (Brizio et al., 2004, 2008). Rat liver mitochondrial matrix gave two fluorescent native bands corresponding to the two covalently flavinylated mitochondrial enzymes sarcosine dehydrogenase and Me_2_GlyDH (Figure 7B, lane 9). The native Me_2_GlyDH was eluted as a fluorescent band at 200 mM NaCl (DEAE200) following DEAE-Sephacel chromatography of mitochondrial matrix. Immunoblotting analysis of DEAE fractions carried out with anti-Hsp60 antibodies revealed that Hsp60 was mainly recovered in the DEAE200 fraction and was completely absent in materials eluted at lower ionic strength (50 mM NaCl, DEAE50). This confirms that mtFSF differs from the matrix chaperone Hsp60 (Brizio et al., 2000). Thus, a matrix component other than Hsp60 might assist flavinylation of apo-Me_2_GlyDH. Instead, we gathered immunoblotting evidence of the Hsp10 co-chaperone being present in the DEAE50 fraction together with the naturally occurring FADS (not shown).

In Figure 7A, to search for possible Me2GlyDH interactors during flavinylation reaction, “re-binding” experiments were performed Briefly, His-Me_2_GlyDH was re-isolated on Ni-chelating Sepharose after a flavinylation step (Brizio et al., 2013). UV fluorescence measurements (compare lanes 1–4, and histogram in the inset) clearly confirm that mitochondrial matrix is able to stimulate apo-Me_2_GlyDH flavinylation (Brizio et al., 2013). This stimulation is observed with the DEAE50 fraction, but not with the DEAE200 one. Both the chaperone Hsp60 and the co-chaperone Hsp10 interact with 6-His-Me_2_GlyDH, as revealed by immunoblotting analysis (Figure 7), therefore they both are expected to participate in the flavinylation machinery. Therefore, the finding that the matrix flavinylation stimulating activity elutes in the same chromatographic fraction that contains mtFADS (Brizio et al., 2013), is prompting us to propose that—besides synthesizing the cofactor—FADS provide a chaperoning activity during Me_2_GlyDH biogenesis.

**Figure 8:**
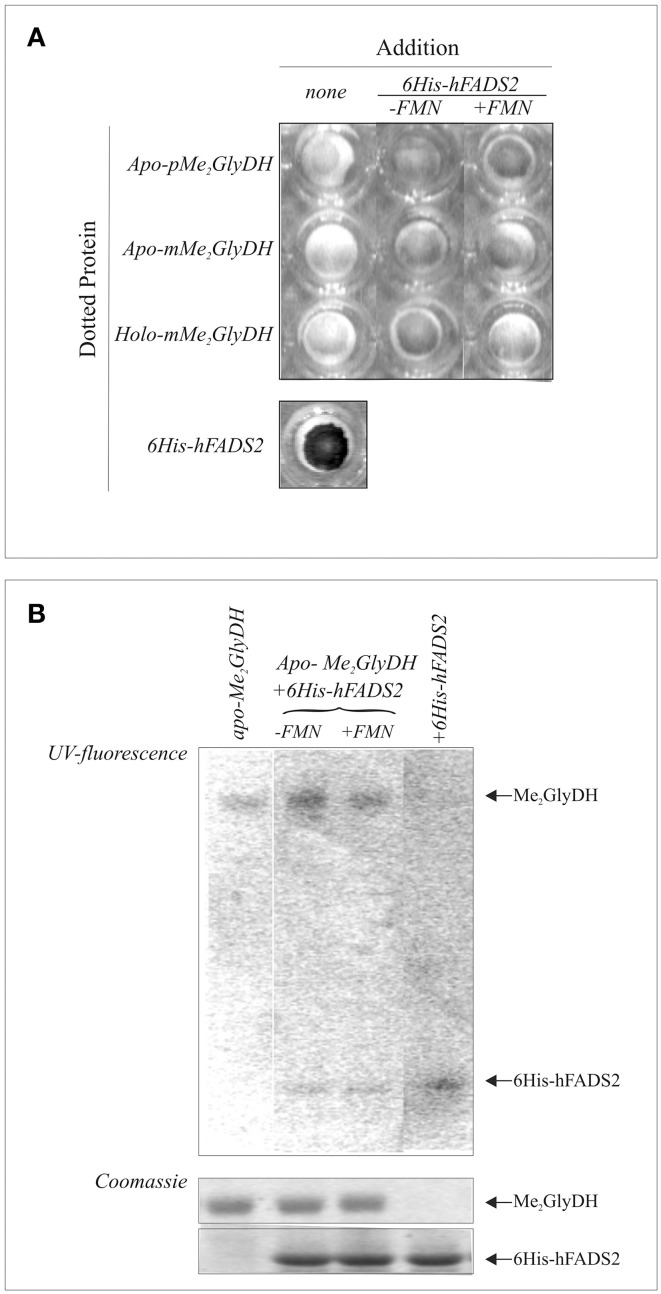
**Evidences of physical interaction and cofactor release from hFADS to the client Me_2_GlyDH**. In **(A)** purified either precursor (p) or mature (m) form of apo- and holo-Me_2_GlyDHs (1 μg each) were dotted onto a nitrocellulose membrane and incubated with purified recombinant human 6His-FADS2 in the presence of 5 mM ATP and 5 mM MgCl_2_. Where indicated 20 μM FMN was added. After 30 min incubation at 37°C the membrane was washed and probed with an anti-hFADS antiserum. In **(B)** purified recombinant apo-pMe_2_GlyDH (1 μg) was incubated at 37°C in the presence or absence of recombinant 6His-hFADS2 (3.3 μg) in 40 mM Hepes buffer pH 7.4 containing 5 mM ATP and 5 mM MgCl_2_. FMN (20 μM) was added where indicated. As a control, 6His-hFADS2 (3.3 μg) was incubated in the same conditions, but in the absence of apo-pMe_2_GlyDH. After 30 min incubation, protein were denatured with the addition of sample buffer, boiled at 95°C and analyzed by SDS-PAGE. The flavin fluorescence of proteins was visualized by UV irradiation of the unstained gel soaked in 10% acetic acid. Proteins were then stained with Coomassie Brilliant Blue.

To further demonstrate that recombinant hFADS physically interacts with the Me_2_GlyDH client, protein-protein interaction was analyzed by dot-blot experiments in which the purified recombinant Me_2_GlyDH (1 μg) was dotted onto the membrane and incubated with purified recombinant human 6His-hFADS2 in the presence of ATP (5 mM) and, when indicated, of FMN (20 μM). After 30 min incubation at 37°C the membrane was washed and probed with an anti-hFADS antibody. These experiments, reported in Figure 8A, indicated that FADS interacts with both the precursor (pMe_2_GlyDH) and mature (mMe_2_GlyDH) form of the acceptor protein.

To test whether direct transfer of FAD to the client protein, purified recombinant apo-pMe_2_GlyDH was incubated in the presence of ATP with purified recombinant hFADS, that binds tightly 1 mol FAD/mol protein. After 30 min incubation, proteins were analyzed by SDS-PAGE, to follow holoenzyme formation. The unstained gel was analyzed to detect flavin fluorescence prior to protein staining. As shown in Figure 8B, hFADS loses its fluorescence upon incubation with apo-Me_2_GlyDH while simultaneously promoting the flavinylation of apo-Me_2_GlyDH. The yield in flavinylated Me_2_GlyDH increased when FMN was added together with ATP.

Based on these and our previous results, we propose that mitochondrial FADS, besides synthesizing FAD, acts in mitochondria as a “*FAD chaperone*” recognizing nascent apo-flavoproteins and promoting their flavinylation.

## Conclusion

Experiments described here confirmed the nuclear localization of FADS in human cells and demonstrate a direct interaction between the FAD forming machinery and the nuclear client flavoprotein LSD1, thus shading light on the possible role of flavin cofactor homeostasis in epigenetic control.

Using the mitochondrial apo-flavoenzyme Me_2_GlyDH, we observed a direct protein-protein interaction and the cofactor transfer from the donor to the apo-protein acceptor, occurring in a sort of “FAD-chaperoning” action played by hFADS *per se*. The role of Hsp60/Hsp10 and a possible control exerted by the redox status of the cofactor during its transfer from FADS to substrate proteins is an interesting matter of future investigation.

**Figure 9:**
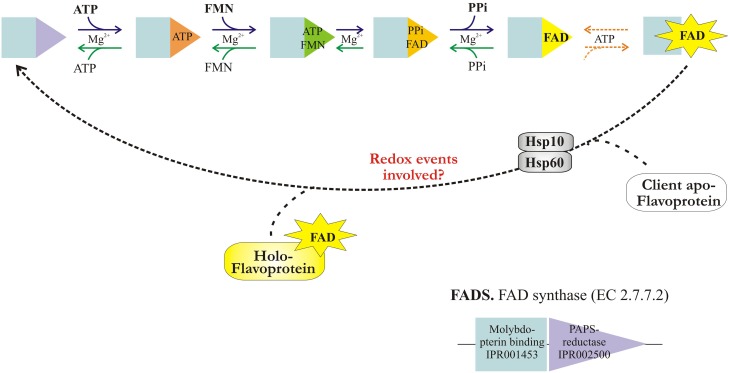
**The catalytic cycle of FAD synthesis and release by 6-His-hFADS2**.

The ordered bi-bi mechanism of FAD synthesis together with the proposal of a flavinylation pathway involving hFADS are presented in Figure 9.

### Conflict of interest statement

The authors declare that the research was conducted in the absence of any commercial or financial relationships that could be construed as a potential conflict of interest.

## Abbreviations

Rf: riboflavin
RFK: riboflavin kinase
FADS: FAD synthase
hFADS: human FAD synthase
hFADS1: human FAD synthase isoform 1
hFADS2: human FAD synthase isoform 2
FLAD1: FAD synthase gene
D-AAO: D-amino acid oxidase
LSD1: Lysine specific demethylase 1
Me_2_GlyDH: dimethylglycine dehydrogenase
mtFSF: mitochondrial flavinylation stimulating factor
PPi: pyrophosphate
LDH: lactate dehydrogenase.
